# Ferroptosis in Human Diseases: Fundamental Roles and Emerging Therapeutic Perspectives

**DOI:** 10.3390/antiox14121411

**Published:** 2025-11-26

**Authors:** Ilaria Artusi, Michela Rubin, Giovanni Cravin, Giorgio Cozza

**Affiliations:** 1 Department of Molecular Medicine (DMM), University of Padua, Via Ugo Bassi 58/B, 35131 Padua, Italy; ilaria.artusi@unipd.it (I.A.); michela.rubin@studenti.unipd.it (M.R.); giovanni.cravin@studenti.unipd.it (G.C.); 2 Department of Pharmaceuticals and Pharmacological Sciences (DSF), University of Padua, Via Marzolo 5, 35131 Padua, Italy; 3 Biostructures and Biosystems National Institute (INBB), Via dei Carpegna 19, 00165 Rome, Italy

**Keywords:** ferroptosis, lipid peroxidation, GPx4, Nrf2, FSP1, lipid radical scavengers, cancer, neurodegenerations, ischemia–reperfusion injury

## Abstract

Ferroptosis is a novel iron-sensitive subtype of regulated cell death (RCD), persisting under extreme lipid peroxidation and iron/redox imbalances. Unlike apoptosis, necroptosis, and pyroptosis, ferroptosis is a signaling-driven process mediated through iron metabolism imbalance, polyunsaturated fatty acid (PUFA) exceeding oxidation, and defects in its protective systems like Xc-/GSH/GPx4. Specifically, this review establishes that iron-driven ferroptosis is a central underlying pathomechanistic factor in a broad range of human diseases. Significantly, whether its modulation is therapeutic, it is entirely conditional on the specific disease context. Thus, its induction can provide a promising antidote for destructive cancer cells when conjoined with immuno-therapies to boost anticancer immunity. Conversely, iron-mediated ferroptosis suppression is a key factor in countering destructive changes in a whole range of degenerative and acute injuries. Current therapeutic approaches include iron chelators, lipid oxidation inhibitors, GPx4 activators, natural and active compounds, and novel drug delivery systems. However, against all odds and despite its intense therapeutic promise, its translation into a practical medicinal strategy faces many difficulties. Thus, a therapeutic agent specifically focused on its modulation is still lacking. The availability of selective biologic markers is a concern. The challenges in the direct pathologic identification of ferroptosis in a complex in vivo systemic scenario remain. Current avenues for its future development are pathogen infections, the discovery of novel regulating factors, and novel approaches to personalized medicine centered on its organ-level in vivo signatures.

## 1. Introduction

Regulated cell death (RCD) is an essential biological process that safeguards tissue homeostasis by eliminating compromised or superfluous cells [1]. Although apoptosis was the first form of RCD to be characterized [2], subsequent research has unveiled a diverse landscape of programmed cell demise, including distinct modalities such as necroptosis [3] and pyroptosis [4]. Among these, ferroptosis has recently emerged as a unique, iron-dependent pathway of cell death, mechanistically and morphologically divergent from other known forms of RCD [5].

The biochemical hallmark of ferroptosis is the iron-catalyzed, non-enzymatic peroxidation of polyunsaturated fatty acids (PUFAs) within cellular membranes. This relentless process culminates in a catastrophic loss of membrane integrity and eventual cell rupture [5]. At its core, ferroptosis is a consequence of a fatal collapse in the cell’s management of iron and redox homeostasis. The true intrigue of this process, however, lies in its inherent duality. Although described as an “essentially spontaneous and uncatalyzed chemical process” [5], this vulnerability is paradoxically held in check by a sophisticated network of metabolic pathways, most notably the glutathione-dependent lipid peroxide repair system catalyzed by glutathione peroxidase 4 (GPx4) [6].

The delicate balance between an inexorable chemical reaction and its intricate biological regulation underscores the complexity of cell fate decisions. Biological systems have evolved robust mechanisms to defend against such intrinsic chemical threats. Disease states often arise not from a single defect, but from a failure of these defenses. Indeed, pathology can manifest when biological control mechanisms are overwhelmed, allowing the spontaneous peroxidative process to dominate, or when critical regulatory nodes are actively dysregulated. Grasping this dynamic is crucial for both unraveling the etiology of numerous diseases and designing novel therapeutic strategies.

In recent years, a surge of research has illuminated the fundamental involvement of ferroptosis across a wide spectrum of human pathologies [7]. Its role has been implicated in the progression of various types of cancer [7], neurodegenerative disorders such as Alzheimer’s and Parkinson’s disease [8], as well as a range of cardiovascular [9], renal [10], and hepatic conditions [11]. This growing focus is shifting the scientific perspective from simple observation to a deeper understanding of ferroptosis as an active participant in disease pathogenesis.

This review, therefore, aims to reframe the discussion, moving beyond merely cataloging the presence of ferroptosis to a robust exploration of its role as a central driver of disease. The language in the scientific literature, which consistently refers to it as a “key factor in pathogenesis”, a “central contributor”, or having a “fundamental role”, underscores its distinction from a passive, secondary effect. These descriptions strongly suggest that ferroptosis is not merely a symptom of tissue damage but an active mechanism that directly fuels disease progression. This paradigm shift has profound implications for therapeutic innovation. By recognizing ferroptosis as a driver rather than a consequence, we can unlock novel treatment strategies aimed at fundamentally altering the course of a disease, rather than just alleviating its symptoms.

## 2. Molecular Underpinnings of Ferroptosis: Core Mechanisms and Regulatory Pathways

### 2.1. Iron Metabolism Dysregulation

**Figure 1:**
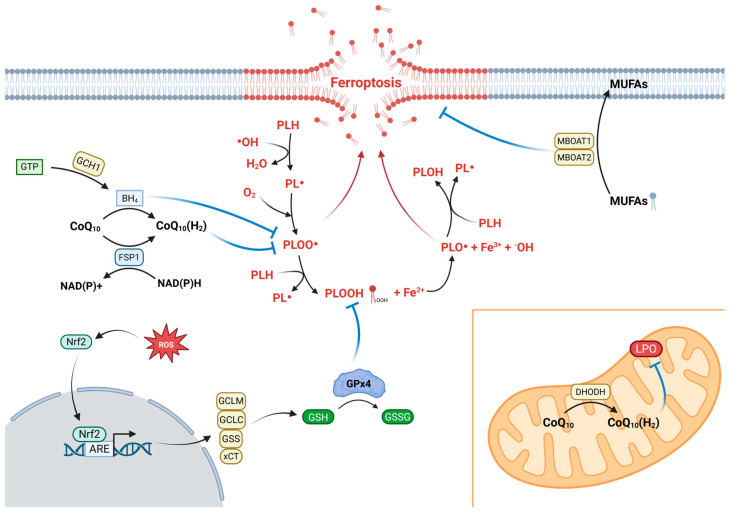
The multi-layered antioxidant defense network against ferroptosis. Ferroptosis is a form of regulated cell death driven by iron-dependent LPO (pathways in red). The process is initiated when a hydroxyl radical (HO^•^) attacks a polyunsaturated fatty acid (PUFA) within a phospholipid (PLH), generating a lipid radical (PL•). This radical enters the propagation phase, reacting with O_2_ to form a lipid peroxyl radical (PLOO•). PLOO• subsequently attacks an adjacent PLH, creating a lipid hydroperoxide (PLOOH) and a new PL•. This cascade is amplified by ferrous iron Fe^2+^, which catalyzes the decomposition of PLOOH into highly reactive alkoxyl radical (PLO•). Cells employ a sophisticated, multi-layered defense system (pathways in blue) to counteract this process: the canonical pathway relies on GPx4/GSH, while the GPX4-independent axes are represented by FSP1/CoQ_10_, GCH1/BH4, DHODH and MBOATs. Created in BioRender. Smith, J. (2025). BioRender.com/c248457.

Iron is a master of duality, a critical component for vital physiological processes like oxygen transport, and a potent catalytic agent for cell toxicity. The property that makes iron an extremely valuable metal for physiological functions is its ability to sustain a dual oxidation state: ferrous (Fe^2+^) and ferric (Fe^3+^). However, this same chemical property makes iron a key player in the Fenton reaction (Fe^2+^ + H_2_O_2_ → Fe^3+^ + HO^•^ + OH^−^), which generates highly destructive hydroxyl radicals (HO^•^), the central initiators of the lipid peroxidation (LPO) chain reaction that defines and drives ferroptosis [5] (Table 1, Figure 1).

To mitigate this inherent risk, cells have evolved a sophisticated and tightly controlled system to maintain iron homeostasis [12]. The regulation of intracellular iron is a precisely orchestrated interplay between import, export, and storage. Iron influx is mediated by two principal pathways. The first involves Transferrin Receptor 1 (TFRC), the gatekeeper for the uptake of transferrin-bound iron. Following endocytosis of the TFRC-transferrin complex, iron is released within the endosome and transported into the cytosol by Divalent Metal Transporter 1 (DMT1) [13,14,15]. DMT1 also facilitates a second pathway: the direct import of non-transferrin-bound ferrous iron (NTBI) across the plasma membrane [12,15]. Upregulation of TFRC or DMT1 expression expands the cell’s labile iron pool (LIP), profoundly sensitizing it to ferroptotic insults [12,16]. Conversely, iron efflux is controlled by the sole known cellular iron exporter, Ferroportin (FPN1). The functional activity of FPN1 is a critical determinant of ferroptosis resistance; its downregulation or degradation traps iron intracellularly, creating a potent pro-ferroptotic state [17]. This protein is under the systemic control of the hepatic peptide hormone hepcidin, which binds FPN1 to induce its internalization and degradation. Hepcidin thus acts as a master switch, locking iron within cells and increasing tissue-level sensitivity to ferroptosis [18] (Table 1).

To handle excess iron that might otherwise fuel the Fenton reaction, the multi-subunit protein complex ferritin functions as a secure intracellular vault, sequestering thousands of iron atoms in a non-reactive form and acting as a crucial defense against ferroptotic stress [19]. This stored iron, however, can be rapidly weaponized through ferritinophagy, the selective autophagy-mediated degradation of ferritin. This process is primarily initiated by the cargo receptor Nuclear Receptor Coactivator 4 (NCOA4), which delivers ferritin to lysosomes [19,20]. Within the acidic lysosomal environment, the ferritin cage is dismantled, flooding the LIP with highly reactive Fe^2+^ and driving the cell toward ferroptosis [20].

This network is further modulated at the post-transcriptional level. Iron-Responsive Element Binding Proteins (IRPs), particularly IRP2, sense intracellular iron levels and bind to Iron-Responsive Elements (IREs) on target mRNAs. This action stabilizes TFRC mRNA while repressing the translation of ferritin and FPN1 mRNAs, thereby coordinating a program that maximizes cellular iron acquisition and bioavailability, effectively priming the cell for ferroptosis [6]. Additional factors, such as Heme Oxygenase-1 (HO-1), can contribute to the LIP by catabolizing heme [21]. In opposition, cytosolic iron chaperones like Poly(rC)-Binding Protein 1 (PCBP1) provide a countervailing protective function by ensuring iron is safely trafficked to its destinations, preventing its illicit participation in redox reactions [22,23]. The coordinated action of these regulators highlights that ferroptosis is not a random event, but a precisely managed process where cellular fate hinges on the delicate and interconnected balance of its iron and redox machinery [5,6] (Table 1).

### 2.2. Lipid Peroxidation Mechanisms

Ferroptosis is executed through the uncontrolled LPO of PUFAs within cellular membranes. This cascade is not a mere consequence of cell death but is its central driver, unleashing a chain reaction that culminates in the formation of cytotoxic secondary products, including reactive aldehydes and lipid electrophiles derived from the decomposition of lipid hydroperoxides (LOOH), which systematically compromise membrane integrity [24] (Figure 1).

The entire destructive process unfolds in distinct phases. It begins with initiation, where a hydroxyl radical (HO^•^), produced by the Fenton reaction from excess labile iron, violently abstracts a hydrogen atom from a PUFA. This creates a carbon-centered lipid radical (L•). The process then enters a vicious, self-sustaining propagation phase. The lipid radical reacts with molecular oxygen to form a lipid peroxyl radical (LOO•), an aggressive species that steals a hydrogen atom from a neighboring PUFA. This generates a lipid hydroperoxide (LOOH) while simultaneously creating a new L•, perpetuating the chain reaction [24,25,26]. This event is then dramatically amplified, as the newly formed LOOH can itself undergo reductive cleavage by ferrous iron, generating a new, highly reactive alkoxyl radical (LO•) and creating a critical branching point that exponentially accelerates the oxidative damage [24] (Table 1, Figure 1).

While this free-radical chemistry is the engine of ferroptosis, its efficiency is profoundly shaped by enzymes that tailor the lipid landscape of the cell. Susceptibility to ferroptosis is first determined by membrane composition. Acyl-CoA synthetase long-chain family member 4 (ACSL4) is a critical gatekeeper; it activates PUFAs like arachidonic acid and adrenic acid for their subsequent incorporation into membrane phospholipids, effectively priming the cell for peroxidation [27,28]. Working in concert with ACSL4, the membrane remodeling enzyme lysophosphatidylcholine acyltransferase 3 (LPCAT3) is responsible for inserting these activated PUFAs into the phospholipid backbone [29]. This synergistic action enriches membranes with highly oxidizable substrates, rendering them exquisitely sensitive to ferroptotic stimuli [30].

When the membrane is primed, iron-containing enzymes known as lipoxygenases (LOXs) can directly and enzymatically mediate LPO. LOXs oxygenate PUFAs in biological membranes, providing a potent pathway to generate lipid hydroperoxides and amplify the oxidative damage. This targeted assault can be facilitated by the scaffold protein phosphatidylethanolamine-binding protein 1 (PEBP1), which binds to 15-lipoxygenase (15-LOX) and guides it to membrane PUFAs, dramatically increasing the efficiency of ferroptosis induction [31] (Table 1).

The terminal products of this relentless PUFA oxidation, such as the highly reactive aldehydes malondialdehyde (MDA) and 4-hydroxynonenal (4-HNE), serve as key biomarkers of lipid peroxidation. More importantly, they are themselves toxic agents that form adducts with proteins and DNA. In the context of ferroptosis, their most critical role is the physical disruption of the membrane bilayer, which leads to increased permeability, loss of electrochemical gradients, and ultimately, cell lysis [32]. However, this lytic event is not a cell-autonomous endpoint. Recent optogenetic studies have demonstrated that ferroptosis can propagate to neighboring cells, which subsequently undergo ferroptotic death themselves [33]. This propagation is not mediated by soluble factors but is a physicochemical process requiring direct, α-catenin-dependent cell–cell contact [33]. The spread is dependent on extracellular iron and is driven by the direct transfer of iron-dependent LPO between the apposed plasma membranes of adjacent cells, effectively transmitting the oxidative damage from the dying cell to its healthy neighbors [33] (Table 1).

**Table 1:** Determinants of ferroptotic sensitivity: the iron and lipid peroxidation axis.

Regulator/Pathway	Role and Mechanism of Action	Impact on Ferroptosis Sensitivity
**Iron Metabolism Dysregulation**		
**Fenton Reaction [5]**	Iron-catalyzed reaction (Fe^2+^ + H_2_O_2_ → Fe^3+^ + HO^•^ + OH^−^) that generates highly reactive hydroxyl radicals (HO^•^)	**Initiates LPO**
**TFRC [13,14,15]**	Primary receptor responsible for the endocytic uptake of transferrin-bound iron.	**Promotes**
**DMT1 [12,15]**	Transports iron from the endosome to the cytosol and directly imports NTBI from outside the cell.	**Promotes**
**FPN1 [17]**	The sole known cellular iron exporter; actively transports iron out of the cytosol.	**Inhibits**
**Hepcidin [18]**	Binds to FPN1, inducing its internalization and degradation. This action traps iron intracellularly.	**Promotes**
**Ferritin [19]**	Safely blocks iron in a non-reactive form.	**Inhibits**
**NCOA4 (Ferritinophagy) [19,20]**	Selective autophagy cargo receptor that targets ferritin for lysosomal degradation, releasing large amounts of Fe^2+^ into the LIP.	**Promotes**
**IRPs [6] **	Iron-responsive element binding proteins. Post-transcriptionally regulate iron homeostasis.	**Promotes**
**HO-1 [21]**	Enzyme that catabolizes heme, releasing free iron and contributing to the LIP.	**Promotes**
**PCBP1 [22,23]**	Cytosolic chaperone that safely binds and traffics iron to its destinations,	**Inhibits**
**LPO Mechanisms**		
**ACSL4 [27,28]**	Activates PUFAs (e.g., arachidonic acid) for membrane incorporation.	**Promotes**
**LPCAT3 [29]**	Inserts ACSL4-activated PUFAs into membrane phospholipids.	**Promotes**
**LOXs [31]**	Directly and enzymatically oxygenate membrane PUFAs to generate lipid hydroperoxides (LOOH).	**Promotes**
**PEBP1 [31]**	Scaffold protein that binds 15-LOX and guides it to membrane PUFAs	**Promotes**
**Cell–Cell Propagation [33]**	α-catenin-dependent cell contact for the transfer of LPO to adjacent cells.	**Promotes (in adjacent cells)**

### 2.3. Antioxidant Defense Systems

To counteract the persistent threat of oxidative damage and prevent the onset of ferroptosis, cells deploy a sophisticated and multi-layered network of antioxidant systems. This is not a static shield but a dynamic and highly regulated in-depth defense, orchestrated by a master transcriptional architect that senses danger and fortifies the cell from within.

At the heart of this proactive defense lies the Nrf2-Keap1 signaling pathway, which acts as the primary command-and-control center for the cellular antioxidant response. The transcription factor Nrf2 (Nuclear factor erythroid 2-related factor 2) is the key effector of this response, yet under homeostatic conditions, its potential is constitutively suppressed by its molecular sentinel and negative regulator, Keap1. This cysteine-rich protein continuously binds to Nrf2, promoting the ubiquitination and subsequent proteasomal degradation of the nuclear factor, thereby maintaining its activity at basal levels [34]. However, upon exposure to oxidative or electrophilic insults, the very triggers of LPO, Keap1 undergoes conformational changes, releasing its grip on Nrf2. This unleashes a powerful counter-offensive: the stabilized Nrf2 translocates to the nucleus, binds to the Antioxidant Response Element (ARE) in the genome, and initiates a coordinated wave of gene expression [35] (Table 2, Figure 1).

This Nrf2-driven transcriptional program is foundational to LPO control, as it orchestrates the reinforcement of the glutathione (GSH)-based defense system. Nrf2’s key targets coordinate the entire synthesis pipeline. It upregulates SLC7A11, encoding the crucial subunit of the cystine–glutamate antiporter System Xc^−^, to secure the necessary cystine supply [36]. Concurrently, Nrf2 drives the expression of the core enzymatic machinery for GSH synthesis. Specifically, it mediates the induction of the genes encoding for the catalytic and modifier subunits of the glutamate–cysteine ligase, which is a rate-controlling step catalyzing the bonding of glutamate and cysteine to produce a γ-glutamylcysteine dipeptide [37]. Moreover, Nrf2 mediates the induction of Glutathione Synthetase (GSS), a catalyst for a final ATP-driven glycine addition to γ-glutamylcysteine during the synthesis of the tripeptide GSH [37] (Table 2, Figure 1). The ultimate effector of this axis is the specialized enzyme GPx4 [38]. As a unique, monomeric selenoprotein, GPx4 possesses the singular ability to navigate the hydrophobic environment of cellular membranes and directly neutralize the LOOH [39,40]. Its catalytic cycle is critically dependent on the GSH pool. GPx4 utilizes two molecules of reduced glutathione (GSH) as the essential cofactor to reduce an LOOH molecule to its benign, non-reactive alcohol form (LOH) [40,41]. During this detoxification, the GSH is oxidized to glutathione disulfide (GSSG), which must then be recycled back to GSH by glutathione reductase (NADPH-dependent) to sustain the defensive effort [42] (Table 2, Figure 1). The central role of this integrated Nrf2/GSH/GPx4 axis is therefore absolute. Its failure, whether through GSH depletion or direct GPx4 inhibition, removes this critical line of defense, serving as a primary executioner of the ferroptotic death program [5]. Notably, Nrf2 also enhances the regeneration of NADPH via the pentose–phosphate pathway [43] and it reconfigures iron metabolism by inducing HO-1 to catabolize pro-oxidant heme [44], simultaneously increasing the expression of ferritin heavy and light chains (FTH1, FTL) to safely sequester the released iron [45] (Table 2, Figure 1).

While the Nrf2-GSH-GPx4 axis forms the primary line of defense, cells are equipped with several crucial GPx4-independent systems that provide functional redundancy and alternative protective strategies. A key parallel pathway is orchestrated by Ferroptosis Suppressor Protein 1 (FSP1), formerly known as AIFM2 [46]. FSP1 functions as a potent lipophilic antioxidant by catalytically regenerating coenzyme Q10 (CoQ10) to its reduced, active form, ubiquinol (CoQ_10_H_2_), using NAD(P)H as a hydride source [47]. Ubiquinol, in turn, functions as a radical-trapping antioxidant that directly intercepts and neutralizes lipid peroxyl radicals (LOO•), thereby breaking the propagation of the peroxidation chain reaction within membranes. This mechanism is fundamentally distinct from that of GPx4, which detoxifies the stable hydroperoxide product (LOOH) rather than the radical propagator [48,49] (Table 2, Figure 1).

One more system that defends the cell from damage is the GCH1-BH4 pathway. GTP cyclohydrolase 1 (GCH1) is the enzyme that controls the rate of the reaction and the one responsible for the synthesis of tetrahydrobiopterin (BH4) from the very beginning. BH4 is a very effective antioxidant produced in the body that inhibits ferroptosis in at least two ways: first, it may directly use the radicals to stop LPO, and, secondly, very importantly, it allows the CoQ10 to be regenerated, thereby functionally coupling its activity to the protective FSP1 axis [50]. An additional layer of defense resides within the mitochondria, involving the enzyme dihydroorotate dehydrogenase (DHODH). Located on the inner mitochondrial membrane, DHODH, a key enzyme in pyrimidine biosynthesis, also contributes to the reduction in the mitochondrial CoQ10 pool [51] (Table 2, Figure 1). This compartment-specific activity provides localized protection against mitochondrial LPO, highlighting the spatially organized nature of the cell’s antioxidant defenses [51]. Beyond these radical-trafficking and detoxification systems, a distinct surveillance mechanism operates “upstream” by modifying the fundamental lipid composition of the membrane itself. This pathway relies on the phospholipid remodeling enzymes membrane-bound glycerophospholipid O-acyltransferase 1 and 2 (MBOAT1 and 2) [52]. The role of these enzymes is to suppress ferroptosis through their preference for incorporating monounsaturated fatty acids (MUFAs) into their phospholipid fraction. This will competitively reduce the level of highly peroxidizable phospholipids containing PUFAs, the primary substrates for LPO. This protective mechanism is notable for being entirely independent of both the GPx4 and FSP1 surveillance pathways. Furthermore, this system is under direct transcriptional control by sex hormones: MBOAT1 is upregulated by the Estrogen Receptor (ER) and MBOAT2 by the Androgen Receptor (AR), directly linking sex-specific hormone signaling to ferroptosis resistance in relevant cancers (Table 2, Figure 1).

Therefore, the integration of iron metabolism, LPO, and antioxidant function defines ferroptosis as a focal regulatory point. The involved factors are highly intertwined and originate as follows: while iron overabundance triggers lipid peroxidation, this process is only lethal in its interaction with dysfunctional GPx4 and FSP1. The self-containing interconnection between iron overabundance and LPO suggests a number of therapeutic weaknesses. This indicates that a therapy that focuses on different points in this network (such as iron chelation, LPO inhibition, and/or support for GPx4 and FSP1) could effectively target this process. Of note is that a multi-target therapeutic strategy could provide enhanced efficacy. Furthermore, the nature of ferroptosis’ core radical chain reactions highlights a fundamental biochemical vulnerability. Once a critical threshold is crossed, the death cascade acquires a chemical autonomy that can become irreversible, marking a physiological point of no return. This inherent weakness, which is exploited in various pathological conditions, underscores the importance of preventative interventions. Conversely, in therapeutic settings such as oncology, intentionally pushing cells beyond this threshold offers a compelling strategy for effectively triggering targeted cell death.

**Table 2:** Mechanisms of ferroptosis resistance: key defensive pathways.

Regulator/Pathway	Role and Mechanism of Action	Impact on Ferroptosis Sensitivity
**The GPX4-Dependent Axis**		
**SLC7A11 [36]**	Nrf2 target. Cystine/glutamate antiporter; imports cystine, the rate-limiting precursor for GSH synthesis.	**Inhibits**
**GCL and GSS [37]**	Nrf2 targets. Key enzymes for GSH synthesis. GCL (GCLC/GCLM) is the rate-limiting enzyme; GSS catalyzes the final step.	**Inhibits**
**GPx4 [39,40,41]**	A unique selenoprotein that utilizes GSH to directly detoxify lipid hydroperoxides (LOOH) into benign lipid alcohols (LOH).	**Inhibits**
**HO-1 and FTH1/FTL [44,45]**	Nrf2 targets. HO-1 catabolizes pro-oxidant heme; ferritin sequesters the released free iron, reducing the LIP.	**Inhibits**
**GPX4-Independent Pathways**		
**FSP1-CoQ10 Axis [46,47,48]**	NAD(P)H-dependent reductase that regenerates coenzyme Q10 (Ubiquinone) to its active antioxidant form, Ubiquinol (CoQ_10_H_2_), which neutralizes the LOO•	**Inhibits**
**GCH1-BH4 Pathway [50]**	Rate-limiting enzyme for the synthesis of Tetrahydrobiopterin (BH4). BH4 acts as an endogenous RTA and promotes CoQ10 regeneration.	**Inhibits**
**DHODH [51]**	An inner mitochondrial membrane enzyme that reduces the mitochondrial CoQ10 pool, providing localized protection against LPO.	**Inhibits**
**Substrate Remodeling**		
**MBOAT1/MBOAT2 [52]**	Incorporate MUFAs into membranes, competitively displacing the highly peroxidizable PUFA.	**Inhibits**

## 3. Ferroptosis in Disease Pathogenesis: A Fundamental Role Across Organ Systems

### 3.1. Cancer

For decades, oncology has focused on inducing apoptosis, a controlled form of cell suicide. However, cancer’s frequent development of apoptosis resistance necessitates the exploration of alternative cell death pathways. In this context, ferroptosis represents an alternative vulnerability that can bypass conventional apoptotic blocks. Understanding how cancer cells regulate their sensitivity to this process is crucial.

**Figure 2:**
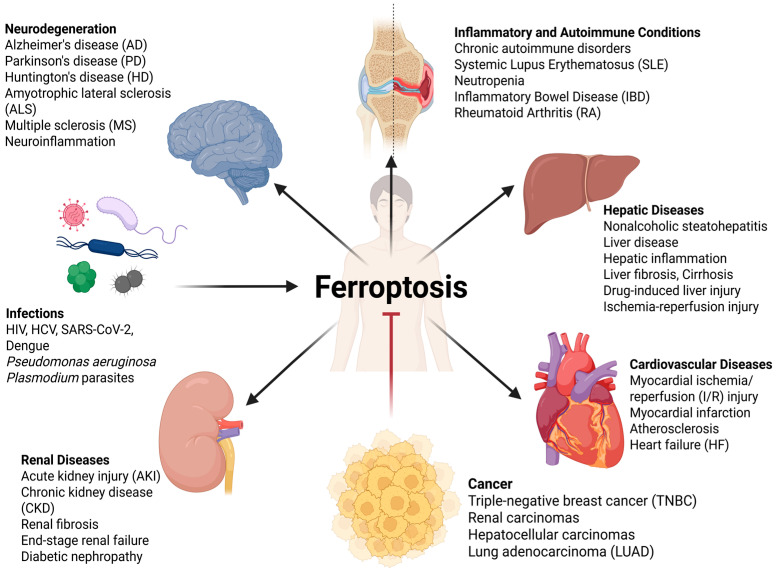
The multifaceted role of ferroptosis in human diseases. Ferroptosis, a distinct form of regulated cell death characterized by iron-dependent lipid peroxidation, is implicated in the pathophysiology of a wide array of human diseases. This figure illustrates its involvement in various organ systems and disease categories. Ferroptosis contributes to neurodegeneration, inflammatory and autoimmune conditions, hepatic diseases, and cardiovascular diseases. Furthermore, various infectious events can activate ferroptosis, while cancers develop strategies to protect themselves from ferroptotic cell death. Created in BioRender. Smith, J. (2025). BioRender.com/c248457.

Cancer cells must actively manage the threat of ferroptosis, which is primarily achieved by maintaining robust antioxidant defenses. The cornerstone of this defense is GPx4, which relies on a steady supply of GSH, synthesized from precursors like cystine, imported via the System Xc^−^ transporter. Cancer cells often fortify this axis to survive the oxidative stress inherent in rapid proliferation and metabolic activity [53,54]. However, the very adaptations that drive malignancy can paradoxically create ferroptotic sensitivity. Cancers driven by RAS-RAF-MEK signaling, for instance, often reprogram their metabolism to accumulate iron, inadvertently priming themselves for ferroptosis [55]. Similarly, cells undergoing epithelial-to-mesenchymal transition (EMT), a process linked to metastasis and therapy resistance, frequently exhibit reduced GPx4 levels, exposing a critical susceptibility [56]. These intrinsic links have identified specific cancer types, including aggressive triple-negative breast cancer (TNBC) [57,58] and certain renal [59] and hepatocellular carcinomas [60], as potentially more reliant on ferroptosis defenses. When the primary GPx4 defense is overwhelmed or bypassed, cancer cells can deploy backup systems like the FSP1 pathway, which represents a significant secondary defense. Notably, FSP1 appears particularly crucial for tumor survival in vivo, protecting against physiological oxidative stress [61]. Indeed, in mouse models of lung adenocarcinoma (LUAD), FSP1 loss triggers LPO and suppresses tumor formation, and high FSP1 expression correlates with poorer patient survival, indicating its biological importance in cancer progression [61,62] (Figure 2).

Furthermore, many aggressive and therapy-resistant cancer cells develop an “iron addiction”. Their heightened metabolic rate, rapid proliferation, and need for epigenetic plasticity demand substantial iron as a cofactor key enzymes involved in DNA synthesis, energy production, and demethylation [63,64]. To meet this heightened iron demand, cancer cells frequently upregulate the iron import proteins TFRC [65,66,67] and DMT1 [68,69]. This elevated iron influx consequently expands the cellular LIP, thereby increasing susceptibility to ferroptosis [70,71]. Crucially, recent research pinpoints the lysosome as the critical initiation site for ferroptosis [72]. This organelle naturally accumulates redox-active iron delivered via processes like autophagy (including ferritinophagy) within an acidic, reducing environment. This creates the ideal conditions, a chemical tinderbox, for iron to catalyze the initial Fenton reactions that trigger the fatal LPO cascade on lysosomal and adjacent membranes [72]. Cancer cells with high iron turnover or specific dependencies, such as certain drug-tolerant persister (DTP) cells often characterized by high CD44 expression, may be particularly susceptible to lysosome-initiated ferroptosis due to their reliance on processing iron through this organelle [72].

Finally, the lytic and inflammatory nature of ferroptosis itself has biological consequences within the tumor microenvironment [73]. The chaotic rupture of ferroptotic cells releases damage-associated molecular patterns (DAMPs) and other signaling molecules [74]. These act as potent immunostimulatory signals, capable of recruiting and activating immune cells, including cytotoxic T lymphocytes, thereby potentially linking this cell death pathway to anti-tumor immunity [75]. This immunogenic aspect represents another layer of how ferroptosis interfaces with cancer biology, influencing the dialogue between the dying tumor cell and the host immune system [73,75].

### 3.2. Neurodegenerative Diseases

The human brain, despite being the center of consciousness and possessing immense computational complexity [76], exhibits unique physiological characteristics that create significant metabolic vulnerabilities [77]. Due to its high oxygen consumption, large catalytic iron reserves, and membranes rich in easily oxidized lipids, the brain is quite prone to oxidative damage like ferroptosis [77]. Initially viewed as a specialized cell death process, ferroptosis is now understood as a major factor in neurodegeneration and a key contributor to Alzheimer’s disease (AD) [78], Parkinson’s disease (PD) [79], Huntington’s disease (HD) [80], amyotrophic lateral sclerosis (ALS) [81], and multiple sclerosis (MS) [82,83].

The brain’s particular structure makes it naturally fragile. While elevated iron levels are important for myelin formation and neurotransmitter synthesis, this high level creates a precarious environment. Normal homeostatic controls break down during aging or disease, and toxic iron accumulates, broadly driving oxidative stress [84]. This issue is worsened by the brain’s special lipid makeup. Its neuron membranes have a lot of PUFAs, especially arachidonic acid and docosahexaenoic acid [85,86]. When iron and PUFAs combine with the brain’s high need for oxygen, this can cause a large amount of LPO. Healthy antioxidant systems, like the GSH/GPx4 axis, cannot stop this damage [87].

Evidence connecting ferroptosis is being found in many neurodegenerative diseases, suggesting distinct pathological contributions in each condition. In AD, ferroptosis seems to be linked to the main signs of the disease: amyloid-beta (Aβ) plaques and neurofibrillary tau tangles. Aβ peptides and hyperphosphorylated tau make iron control worse, promoting the accumulation of redox-active iron [88]. However, the GSH depletion and poor GPx4 function/expression seen in AD come from long-term oxidation caused by Aβ toxicity, neuroinflammation, mitochondrial issues, and the aforementioned iron accumulation [89]. This convergence overwhelms the neuron’s primary defenses, leaving it susceptible to ferroptotic demise [90]. The link is perhaps even more direct in PD, characterized by the progressive loss of dopaminergic neurons in the substantia nigra, a region uniquely rich in iron. Dopamine metabolism itself causes oxidation. Its auto-oxidation and breakdown by monoamine oxidase (MAO) create hydrogen peroxide and other ROS [91]. This, along with high iron levels and membranes full of PUFAs, creates a setting that supports ferroptosis. Critically, a hallmark of PD pathology is a profound depletion of GSH specifically within the substantia nigra, severely crippling the GPx4 antioxidant system and rendering these neurons highly exposed to iron-catalyzed LPO and subsequent ferroptotic death [92,93,94]. In HD, the mutant huntingtin (mHTT) protein causes mitochondrial issues, leading to an increase in ROS creation and energy loss. It also messes with antioxidant responses, causing poor GSH synthesis and turnover [95], while altering iron metabolism in affected brain regions [96]. This combination of increased oxidative load and compromised GSH-GPx4 defenses creates a state of heightened vulnerability to ferroptosis in the striatum [80]. A similar condition of oxidative stress proves fatal for the motor neurons in ALS, where factors like glutamate excitotoxicity [97], iron accumulation [98], and GSH depletion [99] create a perfect storm that these specialized cells cannot survive [81]. In MS, ferroptosis contributes to the disease cycle by killing oligodendrocytes, responsible for myelin production. These cells are naturally iron-rich and depend heavily on GPx4 activity [100]. When myelin is damaged during inflammatory attacks, it releases excess iron. This iron, combined with inflammation, triggers ferroptosis in the already compromised oligodendrocytes, impairing remyelination and worsening neurodegeneration [101] (Figure 2).

This cellular destruction is amplified by a vicious cycle of neuroinflammation. Dying ferroptotic neurons release DAMPs, which act as a siren’s wail, activating nearby glial cells like microglia and astrocytes. Once awakened, these cells unleash a torrent of pro-inflammatory cytokines, further disrupting iron homeostasis and generating more reactive oxygen species, which in turn sensitize neighboring neurons to ferroptosis [102]. This creates a self-perpetuating wave of damage that spreads through neural circuits.

### 3.3. Cardiovascular Diseases

The selective destruction of highly metabolically active cells extends to the cardiovascular system, where ferroptosis is recognized to be a key non-apoptotic executioner in the development and progression of a broad range of cardiovascular diseases (CVDs) [103]. Its role ranges from acute events like myocardial ischemia/reperfusion (I/R) injury [104] and myocardial infarction [105] to chronic, insidious pathologies such as atherosclerosis [106] and heart failure (HF) [107]. The heart’s physiological requirements make it peculiarly susceptible to ferroptosis. As a constantly active organ, the myocardium has a remarkably high density of mitochondria, and therefore has a remarkably high rate of oxygen consumption and a correspondingly high level of basal ROS production [108,109]. This high metabolic rate creates significant vulnerability when combined with two critical factors. First, cardiac cell membranes and mitochondrial structures are highly enriched with readily available PUFAs, especially within key phospholipids like cardiolipin and phosphatidylethanolamines [110]. Second, CVDs frequently disrupt iron homeostasis. In I/R injury, for example, the reperfusion phase introduces oxygenated blood, causing a rapid release of iron from damaged mitochondria [111]. This abrupt increase in the LIP, coinciding with the severe depletion of GSH during ischemia [111], overwhelms the GPx4 defense system. This sequence triggers extensive, uncontrolled LPO, which is considered a major contributor to irreversible myocardial damage following infarction [112]. Similarly, chronic conditions like heart failure are sustained by constant low-level oxidative stress and slow myocardial iron accumulation, leading to the sustained, ferroptosis-driven loss of functional cardiomyocytes and fibrotic remodeling [107]. Even in atherosclerosis, ferroptosis is implicated in the death of foam cells and endothelial cells, potentially contributing to plaque instability and rupture [106] (Figure 2).

### 3.4. Renal Diseases

The kidney, acting as the body’s main filter, works under continuous metabolic stress, which makes it vulnerable to iron-mediated oxidative damage, a key feature of ferroptosis, now recognized as an important factor in both the acute kidney injury (AKI) [113] and the gradual decline seen in chronic kidney disease (CKD) [10,114]. In CKD, ferroptosis has recently emerged as an important mediator in producing renal fibrosis and excessive scarring within this vital organ, which frequently progresses to end-stage renal failure [115]. The kidney is rendered susceptible primarily due to its peculiar tubular epithelial cell biology. These cells possess abundant mitochondria to fuel their demanding reabsorptive functions, making them centers for ROS production and iron handling [116]. In AKI, frequently triggered by ischemia–reperfusion or nephrotoxic insults, these renal cells undergo rapid ferroptotic death due to the combined effects of GPx4 inactivation and a surge in catalytic iron [115,117]. Critically, this acute injury often fails to fully resolve, instead initiating a pathological cascade leading to fibrosis [115]. Ferroptotic tubular cells secrete pro-inflammatory and pro-fibrotic factors, including TGF-β1, which primes interstitial fibroblasts to differentiate into myofibroblasts that produce excessive amounts of extracellular matrix materials (such as collagen) [115]. This scarring can reduce microcirculation in the area causing hypoxia, which additionally induces ferroptosis in adjacent tubular cells, establishing a detrimental positive feedback loop [115]. The efficacy of secondary redundant protective pathways independent of GPx4 activity, including FSP1-CoQ10 and GCH1-BH4 pathways [117], is important for renal protection. The dysfunction and subsequent failure of these redundant protective pathways in CKD render the kidneys exquisitely sensitive to ferroptosis-inducing stimuli [10,114]. The effect is more dramatic in diabetic nephropathy, where hyperglycemia is known to perpetually activate ferroptosis through the enhanced induction of ROS production and altered iron metabolism and enzymatic insertion of easily oxidizable PUFAs within cellular membranes [118,119] (Figure 2).

### 3.5. Hepatic Diseases

The liver plays a central role in both iron storage and lipid metabolism, positioning it as a key organ in the study and impact of ferroptosis. In conditions like nonalcoholic steatohepatitis (NASH), a precursor to more severe liver disease, hepatocytes accumulate excessive lipids (steatosis) [120]. This lipid overload, particularly involving PUFAs, provides abundant substrates for LPO [121]. In addition, related mitochondrial dysfunction [122,123] and endoplasmic reticulum stress [124,125] produce ROS, creating favorable conditions for ferroptosis [126,127]. The subsequent ferroptic cell death of hepatocytes leads to the release of inflammatory mediators. In this scenario, inflammatory hepatopathy facilitates the development of hepatic fibrosis and subsequent cirrhosis [128,129]. Similarly, in acute settings such as drug-induced liver injury (e.g., acetaminophen toxicity) or ischemia–reperfusion insults in organ transplantation, the rapid depletion of GSH compromises the GPx4 antioxidant defense axis, leading to widespread ferroptosis-driven hepatocyte death [130,131,132]. Conversely, this cell death mechanism can be therapeutically leveraged. In liver fibrosis, activated hepatic stellate cells (HSCs) are the primary source of the excessive collagen deposition that leads to scarring. Importantly, activated HSCs exhibit increased susceptibility to ferroptosis compared to quiescent HSCs or hepatocytes [133,134]. This differential sensitivity suggests a potential therapeutic strategy: selectively inducing ferroptosis in activated HSCs to reduce fibrosis while sparing healthy liver tissue [134] (Figure 2).

### 3.6. Inflammatory and Autoimmune Conditions

Inflammation is a vital physiological process but can contribute to pathologies if dysregulated. Ferroptosis has recently emerged as one of the key mediators that contribute to cell death in autoimmune-inflammatory disorders [135]. The complex interplay between inflammation and ferroptosis is bidirectional and often cyclic [136]: inflammatory mediators can trigger and/or prime cells for ferroptosis and vice versa. In addition to this vicious cycle, inflammatory cell death through ferroptosis can release a number of mediators that contribute to enhanced inflammatory signaling. Specifically, ferroptosis is associated with the release of DAMPs like high-mobility group box 1 (HMGB1), which can further amplify the inflammatory response by activating pathways including the NLRP3 inflammasome and the cGAS–STING axis, thereby perpetuating a detrimental feedback loop [137,138]. The pathways linking inflammation to ferroptosis induction are becoming increasingly elucidated. Pro-inflammatory cytokines play a direct role; for instance, tumor necrosis factor-alpha (TNF-α) can increase cellular sensitivity [139], while interferon-gamma (IFNγ), a key T cell cytokine, actively fosters ferroptosis by transcriptionally downregulating the SLC7A11 subunit of System Xc^−^, thus limiting GSH synthesis, which compromises GPx4 function [140] (Figure 2). This disruption of the GPx4/GSH axis is a pivotal node in both inflammatory and infectious contexts. Chronic inflammation also often elevates hepcidin, promoting iron sequestration in cells like macrophages, which increases the LIP and creates a pro-ferroptotic environment [141,142]. This is exploited by pathogens; for example, HIV upregulates hepcidin to degrade ferroportin, increasing intracellular iron and vulnerability to Fenton reaction-mediated damage [143]. Similarly, viruses like SARS-CoV-2 and Dengue can also weaken a host’s antioxidant protection by reducing GSH [144,145] or inhibiting GPx4 [146] (Figure 2). Some pathogens show advanced manipulation skills. For example, the Gram-negative bacterium *Pseudomonas aeruginosa* causes ferroptosis in bronchial epithelial cells by using its secreted lipoxygenase (pLoxA) to create pro-ferroptotic signals from host PUFAs, while also starting chaperone-mediated autophagy (CMA) to break down GPx4 [147,148] (Figure 2). However, this pathogenic role is not universal; ferroptosis can also function as a host defense, as ferroptotic signaling in hepatocytes controls liver-stage Plasmodium parasites [149], and its induction via fatty acid desaturase 2 (FADS2) can inhibit HCV replication [150] (Figure 2). This context-dependent duality defines its role in chronic autoimmune disorders. In Systemic Lupus Erythematosus (SLE), IFN-α inhibits GPx4 expression through CREMα induction, promoting ferroptosis of neutrophils and resulting in neutropenia [151]. In Inflammatory Bowel Disease (IBD), ferroptosis of intestinal epithelial cells caused by dysfunctional GPx4 and heightened ACSL4 protein level damages the mucosal barrier [152]. However, in Rheumatoid Arthritis (RA) and Multiple Sclerosis (MS), the therapeutic strategy is inverted: while ferroptosis of structural cells in RA is detrimental, the selective induction of ferroptosis in pathogenic RA-Fibroblast-like Synoviocytes (RA-FLS) reduces synovitis [153] (Figure 2). Similarly, in MS, pathogenic CD4+ T cells evade ferroptosis by upregulating GPx4 to enhance their inflammatory survival, establishing a scenario where therapeutic ferroptosis induction is required to eliminate the pathogenic cellular pool [83] (Figure 2). This complex, cell-type-specific dependency dictates that effective clinical translation must move beyond general inhibition to employ precise modulation strategies that either protect vulnerable cells or selectively eliminate pathogenic populations.

## 4. Emerging Therapeutic Strategies and Clinical Translation

The expanding understanding of ferroptosis has opened new avenues for therapeutic design. The capacity to precisely modulate this potent cell death pathway has facilitated the development of a diverse spectrum of interventions, ranging from targeted small molecules to advanced biological delivery systems. The therapeutic objective is fundamentally dichotomous: either to induce ferroptosis for the targeted elimination of pathological cells or to inhibit it to protect healthy tissues from damage, contingent upon the specific disease context.

### 4.1. Therapeutic Induction of Ferroptosis: A Weapon Against Malignancy

The therapeutic induction of ferroptosis aims to weaponize the metabolic vulnerabilities inherent in cancer cells. Foundational strategies include direct GPx4 inhibition using small molecules like RSL3 [154,155,156], ML162 [157] or FIN56 [158], thereby triggering lipid peroxide accumulation. Alternatively, GSH depletion strategies employ agents such as erastin [159,160] or the FDA-approved drug and multi-kinase inhibitor sorafenib [161,162] to block the System Xc^−^ antiporter, cutting off the supply of cystine required for GSH synthesis and thus starving GPx4 of its essential cofactor. However, sorafenib’s action is often considered less potent or specific for inducing ferroptosis, potentially due to its pleiotropic effects [163] (Table 3). Expanding the clinical repertoire, APR-246 (eprenetapopt) has emerged as a dual-action therapeuticparticularly effective in acute myeloid leukemia (AML) and solid tumors. While primarily developed to reactivate mutant p53, APR-246 has been shown to potently induce ferroptosis by disrupting the GSH/GPx4 antioxidant axis; furthermore, it triggers ferritinophagy, thereby increasing the labile iron pool and fueling lethal lipid peroxidation [164] (Table 3)

Beyond targeting the primary GPx4/GSH axis, therapies can focus on backup antioxidant pathways. The pharmacologic inhibition of FSP1, which generates the radical-trapping antioxidant ubiquinol independently of GSH, has shown preclinical promise, particularly in lung cancer models where FSP1 appears crucial for in vivo tumor survival [165,166]. Another strategy involves targeting DHODH, an enzyme linked to mitochondrial CoQ10 metabolism and ferroptosis defense. Indeed, the DHODH inhibitor brequinar has been shown to selectively suppress the growth of tumors with low GPx4 expression by inducing ferroptosis [167] (Table 3). Furthermore, combining brequinar with sulfasalazine, an FDA-approved drug known to induce ferroptosis by downregulating the expression of Nrf2 [168], synergistically triggers ferroptosis and effectively suppresses the growth of tumors with high GPx4 expression [167] (Table 3). Similarly, targeting the MBOAT1/2 pathway, which remodels lipids to reduce peroxidizable substrates, represents another GPx4/FSP1/DHODH-independent approach [169]. Cancer’s frequent “iron addiction” presents another avenue; iron overload strategies seek to manipulate iron metabolism, intentionally increasing the LIP to enhance iron-catalyzed Fenton reactions and LPO. This could involve targeting iron import proteins like TFRC [67] and DMT1 [170] or CD44-mediated processes [72]. Recognizing the lysosome as a key initiation site due to its accumulation of redox-active iron has led to novel strategies, such as developing agents like the phospholipid degrader fentomycin-1, which is specifically activated within the lysosome to trigger ferroptosis, proving effective against therapy-resistant DTP cells [72] (Table 3).

Furthermore, the immunogenic nature of ferroptosis opens the possibility of combination with immunotherapy. The release of DAMPs during ferroptotic cell death can recruit and activate T cells, potentially converting “cold” tumors into “hot” ones, thereby synergizing with immune checkpoint inhibitors (ICIs) like anti-PD-1 or anti-CTLA-4 monoclonal antibodies [171]. For instance, inhibition of the receptor tyrosine kinase TYRO3 has been shown to promote ferroptosis and sensitize tumors to anti-PD-1 therapy [172]. This represents a significant conceptual evolution: ferroptosis is not merely a cell-killing mechanism but an active signaling event that can prime or enhance the immune system’s attack on cancer cells (Table 3).

Finally, the complexity of these pathways and the potential for resistance, such as adaptive changes involving CD44 downregulation following fentomycin-1 exposure [173], highlights the critical need for the development of biomarkers. Identifying signatures based on metabolic profiles, the expression levels of key regulators like FSP1, or lipid composition will be essential for patient stratification and guiding effective, personalized ferroptosis-based therapies.

**Table 3:** Therapeutic inducers of ferroptosis.

Class	Compound	Mechanism of Action
**GPx4 Inhibitors**	**RSL3 [154,155,156]**	Direct GPx4 inhibitor.
	**ML162 [157]**	Direct GPx4 inhibitor.
	**FIN56 [158]**	Direct GPx4 inhibitor (promotes degradation).
	**ARP-246 [164]**	P53 activator, induce ferroptosis by disrupting the GSH/GPx4 axis
**GSH Depletors**	**Erastin [159,160]**	Blocks System Xc^−^ antiporter, inhibiting cystine uptake.
	**Sorafenib [161,162,163]**	Multi-kinase inhibitor; also blocks System Xc^−^ antiporter.
	**Sulfasalazine [168]**	Induces ferroptosis (Nrf2 downregulation).
**GPx4-Independent Pathway Inhibitors**	**Brequinar [167]**	DHODH inhibitor (targets mitochondrial CoQ10 metabolism).
**Lysosome-Targeting Agents**	**Fentomycin-1 [72]**	Phospholipid degrader specifically activated within the lysosome.
**Immunotherapy (Combination)**	**Anti-PD-1 mAb [171]**	Immune checkpoint inhibitor (synergizes with ferroptosis).
	**Anti-CTLA-4 mAb [171]**	Immune checkpoint inhibitor (synergizes with ferroptosis).

### 4.2. Therapeutic Inhibition of Ferroptosis: A Shield for Degenerative Disease and Injury

In stark contrast, the therapeutic goal in degenerative diseases and acute injuries is to staunchly inhibit ferroptosis to preserve tissue and function [12]. This strong mechanistic link confirms that ferroptosis is a critical therapeutic target across a surprisingly broad range of pathologies, with strategies focused on disarming the main drivers of the cascade. The most direct approach involves meticulous iron management and chelation, a strategy hinging on the deployment of agents capable of sequestering the catalytically active iron that fuels LPO chain reaction [12].

A quartet of such molecules, deferoxamine (DFO), deferiprone (DFP), deferasirox (DFX), and dexrazoxane (DZR), have emerged as powerful, albeit distinct, tools for this purpose, and their application now extends far beyond their initial indications (Table 4). While DFO remains a classic treatment for systemic iron overload, its utility has been demonstrated in models of acute, localized iron toxicity [174]. In the catastrophic setting of hemorrhagic stroke or traumatic brain injury, for example, where the lysis of erythrocytes unleashes a flood of toxic heme-iron, DFO can mitigate secondary brain injury by scavenging this free radical catalyst [174]. A similar principle applies to acute kidney injury, where DFO confers significant nephroprotection against ischemia–reperfusion damage by curbing ferroptosis in renal tubular cells [175,176].

The true therapeutic frontier, however, may lie with orally available, brain-penetrant agents. DFP, due to its ability to cross the blood–brain barrier, has become a focal point of research not just for Parkinson’s disease, but also for other neurodegenerative conditions defined by iron dyshomeostasis [84]. This includes Friedreich’s Ataxia, a disease directly caused by a defect in mitochondrial iron handling [177,178], and Huntington’s disease, where DFP has shown promise in reducing toxic protein aggregates in preclinical models [80]. Its oral counterpart, DFX, is also being explored beyond systemic iron overload for its potential to quell the iron-driven oxidative stress within the smoldering inflammatory landscape of NASH, aiming to halt its progression to fibrosis [179]. Standing somewhat apart is DZR, the designated cardioprotectant. While its primary mechanism involves topoisomerase II inhibition [180], its function as a powerful iron mop, proven to mitigate myocardial ischemia–reperfusion injury, cements its role as a potent anti-ferroptotic agent [181,182].

Beyond the realm of synthetic pharmacology, nature provides its own sophisticated arsenal of iron-chelating agents, chiefly in the form of plant-derived polyphenols and flavonoids. These phytochemicals are of particular interest due to their pleiotropic effects, combining direct iron sequestration with potent intrinsic antioxidant and anti-inflammatory activities [183]. Epigallocatechin-3-gallate (EGCG) from green tea, for instance, has demonstrated significant neuroprotective capacity in models of Alzheimer’s and Parkinson’s disease, a feat attributed to its ability to cross the blood–brain barrier and quell iron-driven oxidative stress [184,185]. Similarly, curcumin, the bioactive compound in turmeric, functions as a powerful chelator and has shown efficacy in mitigating iron accumulation in both neurodegenerative and hepatic injury models [186,187]. Other widely studied flavonoids, such as quercetin and the stilbenoid resveratrol, exert comparable protective effects in contexts of cardiovascular and renal ischemia–reperfusion injury [188,189], where they inhibit ferroptosis by disarming the catalytic iron at the heart of the damage cascade [190] (Table 4). The therapeutic translation of these natural agents, however, is not without challenges. Significant hurdles in bioavailability, metabolic stability and the standardization of extracts currently limit their clinical application; however, ongoing research into advanced delivery systems, such as nano-formulations, could overcome these limitations. Recent advances underscore the potential of this approach. In one instance, curcumin encapsulated in polymeric nanoparticles was found to possess greater anti-ferroptotic activity, and better retain this activity, in an acute kidney injury model compared to free curcumin [191,192]. Similarly, EGCG coordinated metal–organic networks have been created with stability and targeted delivery properties to protect cells from death due to lipid peroxidation [193,194]. In addition, biomimetic strategies like quercetin-loaded exosomes are being developed as carrier systems to cross physiological barriers and upregulates the Nrf2/GPx4 axis to promote neuroprotective effects [195,196]. Thus, in addition to improving pharmacokinetic properties, these nano-architectures have the potential to precisely target the ferroptotic machinery in damaged tissues.

Orthogonal to the strategy of iron sequestration is a more direct approach: erecting a bulwark to protect lipids themselves using potent, radical-trapping antioxidants (RTAs). This tactic aims to neutralize the destructive cascade at its point of action. The body’s endogenous implementation of this strategy is embodied by Vitamin E, a family of eight related lipophilic compounds (tocopherols and tocotrienols) that constitute the primary physiological RTAs [197] (Table 4). Their mechanism is highly specific: they are not efficient scavengers of the highly reactive species that initiate the oxidative cascade, such as HO^•^ or LO•. Instead, they function as peerless chain-breaking antioxidants, intercepting the lipid peroxyl radicals LOO• that propagate the chain reaction [197,198]. This physiological mechanism, which distinguishes between forms like α-tocopherol as the principal LOO• scavenger and γ-tocopherol as a specialized scavenger for reactive nitrogen species, also defines its critical limitation [197,198]. By focusing solely on propagation, Vitamin E can be overwhelmed in the face of potent oxidative insults where the rate of initiation is too high, proving insufficient to compete with the destructive cascade [198]. This limitation of the endogenous system spurred the development of synthetic RTAs capable of a more comprehensive defense. Prototypical lipophilic sentinels like ferrostatin-1 (Fer-1) and liproxstatin-1 (Lip-1) physically intercalate within cellular membranes, where they act as sacrificial shields, intercepting and quenching both the initiation and the propagation steps of LPO, thus preserving membrane integrity [24,199] (Table 4). The therapeutic potential of these molecules has been validated across a remarkable spectrum of preclinical models, including acute kidney and cardiac ischemia–reperfusion injury [200,201,202], traumatic brain injury [203,204], and neurodegenerative diseases [205,206,207]. However, their clinical translation has been hampered by poor pharmacokinetic profiles, spurring the development of next-generation RTAs with enhanced stability and bioavailability.

A parallel and highly strategic approach is to fortify the cell’s intrinsic cytoprotective machinery, primarily the central GSH/GPx4 axis. This can be achieved by increasing substrate availability, most classically through the administration of the GSH precursor N-acetylcysteine (NAC) [208]. However, a more sought-after goal is the direct pharmacological modulation of the master enzyme itself. The development of small molecules capable of binding to and either stabilizing GPx4 protein or allosterically enhancing its catalytic activity represents a “holy grail” in the field. However, the rational design of protein activators, as opposed to inhibitors, presents a formidable pharmacological hurdle. This challenge is exemplified by the trajectory of ebselen; though once reported as a GPx4 mimetic, its advancement into the clinic ultimately resulted in a series of unsuccessful trials, underscoring the difficulty of achieving true enzymatic gain-of-function [209] (Table 4). Beyond modulating individual defense pathways, an even more encompassing strategy involves activating the master regulator of the antioxidant response, Nrf2 (Table 4). Its powerful, proactive reinforcement of cellular defenses can be triggered by a range of activators, both natural and synthetic. These molecules act as electrophiles that covalently modify critical sensor cysteines on Keap1, releasing Nrf2 to the nucleus where it activates a broad set of genes that build up cellular defenses [210]. Potent natural activators include sulforaphane, an isothiocyanate from cruciferous vegetables [211], and pleiotropic compounds like curcumin and resveratrol, which augment their direct iron-chelating and antioxidant effects with robust Nrf2 induction [212,213]. On the synthetic front, this strategy has achieved significant clinical success. Fumaric acid esters, such as Dimethyl Fumarate (DMF) and its next-generation prodrug Diroximel Fumarate (DRF), are approved therapies for multiple sclerosis that function primarily through potent Nrf2 activation [214,215]. More targeted still are molecules like Bardoxolone Methyl and its analog Omaveloxolone, recently approved for Friedreich’s Ataxia, a landmark clinical validation of Nrf2 activation for a pathology intimately linked to ferroptosis [216] (Table 4).

Perhaps the most groundbreaking recent advances have come from elucidating the cell’s sophisticated, GPx4-independent defense systems, which function as a multi-layered, redundant network. The discovery of these pathways has opened entirely new therapeutic avenues. A prominent example is the FSP1/CoQ10/ubiquinol pathway, which regenerates the lipophilic antioxidant ubiquinol (CoQH_2_) [46], therefore offering a powerful, GPx4-parallel defense mechanism [217]. Concurrently, the GCH1-mediated synthesis of BH4 provides a distinct pool of potent radical-trapping antioxidants that protect PUFAs [50]. More recently, the mitochondrial enzyme DHODH was identified as a third defense system, protecting the inner mitochondrial membrane by reducing coenzyme Q [218]. Targeting these pathways, FSP1, GCH1, and DHODH, could offer a sophisticated way to build cellular resilience, not by introducing an external antioxidant, but by activating the body’s own powerful and highly regulated backup machinery against ferroptotic assault.

The central role of ferroptosis in driving pathologies, such as the transition from acute renal injury to irreversible fibrosis, has established it as a prime target for cutting-edge interventions. Looking forward, the field is advancing toward revolutionary therapeutic modalities. One promising avenue is the use of exosome-based gene delivery systems; these natural nanoparticles can be engineered to deliver protective cargo, such as specific microRNAs or GPx4 itself, directly to target cells [219]. In parallel, cell-based therapies using stem cells hold immense potential for neurodegeneration. This strategy is twofold: it aims to provide not only cell replacement but also robust support, as the stem cells can secrete a cocktail of neuroprotective factors or antioxidant key enzymes that shield vulnerable neurons from ferroptotic insults [220].

**Table 4:** Therapeutic inhibitors of ferroptosis.

Class	Compound	Primary Mechanism of Action
**Iron Chelators**	**Deferoxamine (DFO)** **[174,175,176]**	Iron chelator (systemic).
	**Deferiprone (DFP) [80,84,177,178]**	Iron chelator (oral, brain-penetrant).
	**Deferasirox (DFX) [179]**	Iron chelator (oral).
	**Dexrazoxane (DZR)** **[180,181,182]**	Iron chelator; Topoisomerase II inhibitor.
**Natural Products (Polyphenols)**	**EGCG [184,185]**	Natural polyphenol; iron chelator; antioxidant.
	**Curcumin [186,187,212]**	Natural polyphenol; iron chelator; Nrf2 activator.
	**Quercetin [188,190]**	Natural flavonoid; iron chelator.
	**Resveratrol [189,213]**	Natural stilbenoid; iron chelator; Nrf2 activator.
**Radical-Trapping Antioxidants (RTAs)**	**Vitamin E (α-tocopherol) [197,198]**	Physiological RTA (chain-breaking scavenger).
	**Ferrostatin-1 (Fer-1) [24,199]**	Synthetic RTA (blocks LPO initiation and propagation).
	**Liproxstatin-1 (Lip-1) [24,199]**	Synthetic RTA (blocks LPO initiation and propagation).
**GSH/GPx4 Axis Support**	**N-acetylcysteine (NAC) [208]**	GSH precursor.
	**Ebselen [209]**	GPx4 mimetic (organoselenium compound).
**Nrf2 Activators**	**Sulforaphane [211]**	Natural Nrf2 activator (isothiocyanate).
	**Dimethyl Fumarate (DMF) [214,215]**	Synthetic Nrf2 activator (fumaric acid ester).
	**Diroximel Fumarate (DRF) [214,215]**	Synthetic Nrf2 activator (Prodrug of DMF).
	**Bardoxolone Methyl [216]**	Synthetic Nrf2 activator (triterpenoid).
	**Omaveloxolone [216]**	Synthetic Nrf2 activator (triterpenoid).

## 5. Challenges and Future Perspectives in Ferroptosis Research

Despite the rapid ascent of ferroptosis from a cellular curiosity to a central player in pathophysiology, a profound translational gap remains. The field’s foundational tools, such as erastin and RSL3, are invaluable for mapping the molecular pathways, but they are ill-suited for clinical use due to poor pharmacokinetics. They are excellent probes, not viable drugs. This has created a paradox: we have a wealth of mechanistic knowledge but a stark scarcity of clinically approved, direct ferroptosis modulators. While agents like omaveloxolone and DMF have received regulatory approval, their mechanism, activating the master antioxidant regulator Nrf2, represents a broad, indirect reinforcement of cellular defenses rather than a precision strike against a core driver like GPx4. Conversely, edaravone, approved for the treatment of ALS [221], exhibits generic antioxidant properties, but its specific mechanism has not been fully elucidated and appears to straddle a dual role as both a lipid radical scavenger [222] and an activator of the Nrf2 pathway [223,224]. To bridge this void, one promising strategy is drug repositioning: identifying existing, approved drugs that can be repurposed as direct lipid antioxidants. For example, compounds such as the asthma medication zileuton [225], along with seratrodast [226] and formoterol [227], have demonstrated the ability to directly intercept LPO. This approach offers a potential fast-track to the clinic by leveraging established safety profiles, yet it still underscores the lack of a de novo “statin-equivalent”, a bespoke agent specifically designed to potently inhibit a key node in the ferroptosis cascade.

This primary challenge is compounded by two further issues: complex biology and the absence of reliable biomarkers. In virtually no disease, be it stroke, neurodegeneration, or organ failure, does ferroptosis occur in isolation. It is intricately interwoven with apoptosis, necroptosis, and inflammatory signaling. This creates a formidable “attribution problem” for clinical trials: proving that a drug’s benefit stems specifically from inhibiting ferroptosis, rather than a general anti-inflammatory effect, is exceedingly difficult [228]. This ambiguity is exacerbated by a lack of specific biomarkers. Current methods, which rely on invasive tissue sampling to measure LPO products like 4-HNE or MDA, are neither specific to ferroptosis nor practical for routine monitoring. The development of non-invasive, real-time biomarkers, perhaps PET tracers or unique circulating lipid signatures, remains a critical, unmet need [229]. It is noteworthy that in response to these methodological challenges and the rapid expansion of the field, expert recommendations have recently been published. These guidelines aim to increase the robustness and reproducibility of ferroptosis research by outlining state-of-the-art assessment methods, discussing common experimental pitfalls, and providing guidance on validated animal models and small-molecule modulators [230,231].

However, these hurdles do not signal an impasse; rather, they define the exciting frontiers of future research. A primary goal is the development of tissue-specific ferroptosis modulators. Systemically blocking a fundamental cell death process is a high-risk strategy; a drug that protects neurons could inadvertently promote the survival of nascent cancer cells or impair immune function. The pharmacological “holy grail” is therefore to design therapies that selectively modulate ferroptosis only in the target organ, a challenge requiring a deeper understanding of tissue-specific metabolic vulnerabilities.

Another burgeoning frontier is the complex interplay between ferroptosis and the immune system. The immunogenic nature of ferroptotic cell death, driven by the release of DAMPs, presents a fascinating therapeutic dichotomy. In cancer, inducing ferroptosis can serve as an adjuvant to immunotherapy, transforming immunologically “cold” tumors into “hot” ones that are visible to the immune system. Conversely, in chronic inflammatory diseases, blocking the ferroptosis-driven release of DAMPs could be a powerful anti-inflammatory strategy. Finally, the regulatory landscape controlling ferroptosis is proving far more complex than initially envisioned. The role of iron as a “micronutrient battleground” during viral infections, where ferroptosis may function as both a host defense and a pathway exploited by pathogens, offers new paradigms for antiviral therapies, suggesting we are only just scratching the surface of its intricate regulation.

## 6. Conclusions

Ferroptosis has come to the forefront as a fundamental and unique regulated form of cell death that plays an essential role in the pathophysiology of numerous diseases, such as cancer, neurodegenerative disorders, cardiovascular disease, renal and hepatic disease, and inflammatory conditions, among others. The molecular wiring, relating to iron dysregulation, LPO, and reduced antioxidant capacity, remains conservatively maintained throughout all diseases. However, while the nature of ferroptosis and its potential to modulate therapy will be dependent on disease context, a very exciting aspect pertains to research on modulating human immune response via immunogenic cell death.

Even though ferroptosis provides an ample opportunity for novel therapeutic options, there are obstacles in translating this to clinical use due to a lack of available approved drugs, the limitations of biomarkers, and the challenges of isolating its role in a disease-related context in vivo. Future opportunities center upon the development of novel therapies, including natural active compounds and targeted delivery methods, and also addressing poorly explored topic areas, such as ferroptosis’ role in viral infections and novel regulatory mechanisms, among others. Ongoing continued and innovative research will be needed to enable advances in our understanding of ferroptosis and determine potential effective targeted therapeutic strategies for human disease.

As a conclusive remark it should be noted that, despite the exponential growth of the literature and the thousands of reviews published in the last decade, a distinct translational gap persists. Consequently, the significance of the present review lies in looking beyond the current limitations to propose a concrete roadmap for the future. We posit that bridging this void will not come from generic antioxidants, but from a three-pronged strategy: the drug repositioning of safe lipophilic agents (e.g., zileuton), the validation of pleiotropic natural compounds, and the engineering of advanced nano-delivery systems to ensure tissue specificity.

## Data Availability

No new data were created or analyzed in this study. Data sharing is not applicable to this article.

## Abbreviations

The following abbreviations are used in this manuscript:

15-LOX	15-lipoxygenase
4-HNE	4-hydroxynonenal
Aβ	amyloid-beta
ACSL4	Acyl-CoA synthetase long-chain family member 4
AD	Alzheimer’s disease
AKI	acute kidney injury
ALS	amyotrophic lateral sclerosis
AR	androgen receptor
ARE	antioxidant response element
BH4	Tetrahydrobiopterin
CKD	chronic kidney disease
CMA	chaperone-mediated autophagy
CoQ10	coenzyme Q10
CoQH_2_/CoQ_10_H_2_	Ubiquinol
CVDs	cardiovascular diseases
DAMPs	damage-associated molecular patterns
DFO	Deferoxamine
DFP	Deferiprone
DFX	Deferasirox
DHODH	dihydroorotate dehydrogenase
DMT1	divalent metal transporter 1
DMF	dimethyl fumarate
DRF	diroximel fumarate
DTP	drug-tolerant persister
DZR	Dexrazoxane
EGCG	epigallocatechin-3-gallate
EMT	epithelial-to-mesenchymal transition
ER	estrogen receptor
FADS2	fatty acid desaturase 2
Fer-1	ferrostatin-1
FPN1	ferroportin
FSP1	ferroptosis suppressor protein 1
FTH1	ferritin heavy chain
FTL	ferritin light chain
GCH1	guanosine triphosphate cyclohydrolase 1
GCL	glutamate–cysteine ligase
GPx4	glutathione peroxidase 4
GSH	glutathione
GSS	glutathione synthetase
GSSG	glutathione disulfide
HD	Huntington’s disease
HF	heart failure
HMGB1	high-mobility group box 1
HO-1	heme oxygenase-1
HSCs	hepatic stellate cells
IBD	inflammatory bowel disease
ICIs	immune checkpoint inhibitors
IFNγ	interferon–gamma
I/R	ischemia/reperfusion
IREs	iron-responsive elements
IRPs	iron-responsive element binding proteins
L•	lipid radical
Lip-1	liproxstatin-1
LIP	labile iron pool
LO•	alkoxyl radical
LOO•	lipid peroxyl radical
LOOH	lipid hydroperoxides
LOXs	lipoxygenases
LPCAT3	lysophosphatidylcholine acyltransferase 3
LPO	lipid peroxidation
LUAD	lung adenocarcinoma
MAO	monoamine oxidase
MBOAT	membrane-bound glycerophospholipid O-acyltransferase
MDA	malondialdehyde
mHTT	mutant huntingtin
MS	multiple sclerosis
MUFAs	monounsaturated fatty acids
NAC	N-acetylcysteine
NACs	natural active compounds
NASH	nonalcoholic steatohepatitis
NCOA4	nuclear receptor coactivator 4
Nrf2	nuclear factor erythroid 2-related factor 2
NTBI	non-transferrin-bound ferrous iron
PCBP1	poly(rC)-binding protein 1
PD	Parkinson’s disease
PEBP1	phosphatidylethanolamine-binding protein 1
PUFA	polyunsaturated fatty acid
RA	rheumatoid arthritis
RA-FLS	RA-fibroblast-like synoviocytes
RCD	regulated cell death
ROS	reactive oxygen species
RTAs	radical-trapping antioxidants
SLE	systemic lupus erythematosus
TFRC	transferrin receptor 1
TNBC	triple-negative breast cancer
TNF-α	tumor necrosis factor-alpha
